# Beyond ST-Segment Elevation: Recognizing the Aslanger ECG Pattern After Bentall Surgery

**DOI:** 10.1016/j.jaccas.2026.108030

**Published:** 2026-05-06

**Authors:** Rafael Alessandro Ferreira Gomes

**Affiliations:** Division of Cardiology, Pronto-Socorro Cardiológico de Pernambuco (PROCAPE), University of Pernambuco, Recife, Pernambuco, Brazil

**Keywords:** acute coronary syndrome, aortic valve, complication, electrocardiogram, percutaneous coronary intervention

**Figure:**
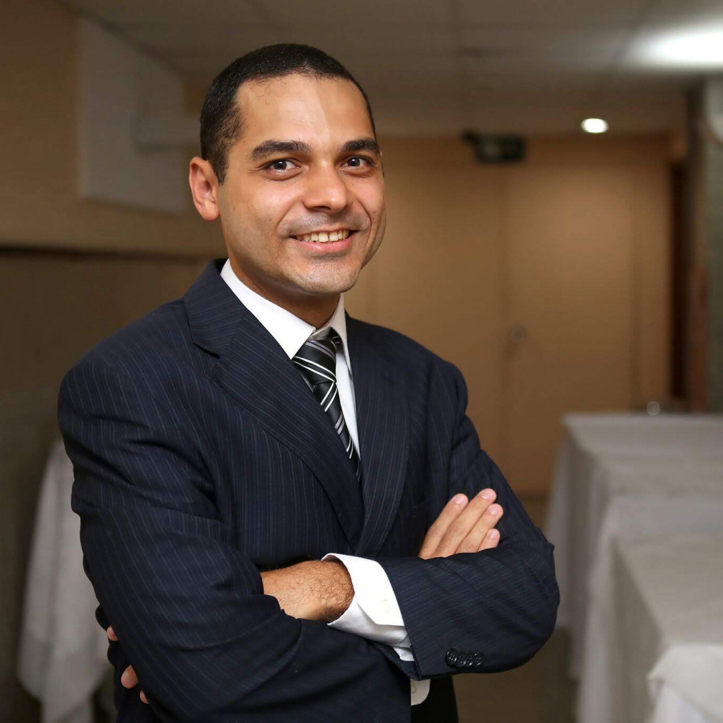

**Visual Summary:**
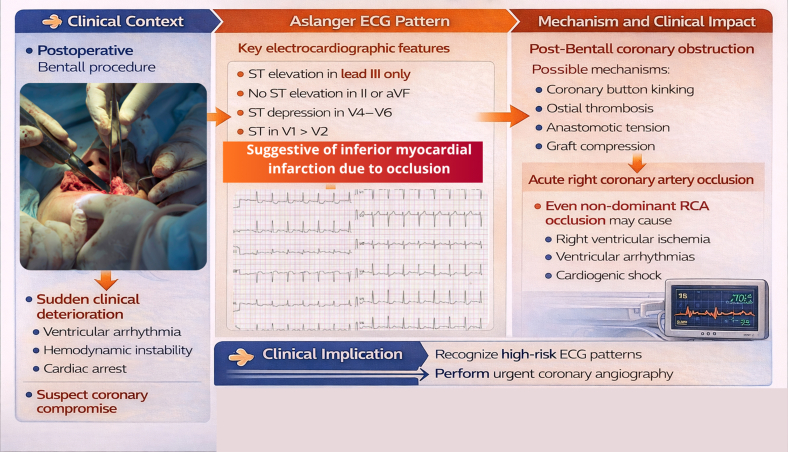
Recognizing the Aslanger ECG Pattern After Bentall Surgery In the postoperative Bentall setting, sudden clinical deterioration should raise suspicion of coronary compromise. The Aslanger ECG pattern suggests inferior myocardial infarction due to acute coronary occlusion. Possible mechanisms include coronary button kinking, ostial thrombosis, anastomotic tension, or graft compression. Even nondominant RCA occlusion may cause right ventricular ischemia, malignant arrhythmias, and cardiogenic shock. Early recognition should prompt urgent coronary angiography and revascularization. ECG = electrocardiogram; RCA = right coronary artery.

The electrocardiogram (ECG) remains one of the most powerful and rapidly accessible diagnostic tools in cardiovascular medicine. Despite major advances in cardiac imaging and biomarker testing, timely recognition of ischemic patterns on ECG continues to guide lifesaving decisions in acute care. Importantly, not all coronary occlusions manifest with the classic criteria for ST-segment elevation myocardial infarction (STEMI). Identifying alternative ECG signatures of coronary occlusion becomes particularly critical in complex clinical scenarios, such as the postoperative period after cardiac surgery.

In this issue of *JACC: Case Reports*, López-Tejada et al^1^ describe a remarkable case of sudden cardiac arrest occurring 3 days after an apparently successful Bentall procedure, ultimately caused by acute ostial occlusion of a nondominant right coronary artery (RCA). Recognition of the Aslanger ECG pattern prompted urgent coronary angiography and successful percutaneous revascularization. This report elegantly illustrates how careful ECG interpretation can rapidly reveal acute coronary occlusion even in diagnostically challenging postoperative circumstances.

Coronary compromise after aortic root surgery is uncommon but potentially catastrophic. The Bentall procedure, first described by Bentall and De Bono in 1968,^2^ involves composite replacement of the aortic valve and ascending aorta, with reimplantation of the coronary arteries into a vascular graft. Although contemporary surgical outcomes are generally excellent, postoperative coronary obstruction remains a recognized complication that may result from coronary button kinking, ostial thrombosis, anastomotic tension, or external compression by the prosthetic graft.^3^

The clinical presentation of coronary obstruction after aortic root surgery can be highly variable, ranging from subtle myocardial ischemia to cardiogenic shock or sudden cardiac arrest. In the early postoperative setting, diagnostic uncertainty is common because several competing etiologies must be considered, including prosthetic valve dysfunction, cardiac tamponade, pulmonary embolism, or postoperative low-output syndrome. Under these circumstances, the ECG often provides the first critical clue pointing toward myocardial ischemia and the need for urgent coronary angiography.

Perhaps the most instructive aspect of the case reported by López-Tejada et al^1^ lies in the recognition of the Aslanger ECG pattern, a recently described constellation of ECG findings associated with acute inferior myocardial infarction. The pattern consists of ST-segment elevation in lead III without corresponding elevation in leads II or aVF, ST-segment depression in the lateral precordial leads (V_4_-V_6_), and an ST-segment level in lead V_1_ that exceeds that in lead V_2_, reflecting a rightward-directed injury vector.^4^

Recognition of such ECG patterns has gained increasing relevance as the conceptual framework of acute coronary syndromes evolves beyond the traditional dichotomy of STEMI and non–ST-segment elevation myocardial infarction (NSTEMI). A growing body of evidence suggests that a significant proportion of patients with acute coronary artery occlusion present without classic ST-segment elevation. Instead, these patients may exhibit alternative ECG signatures—often described as occlusion myocardial infarction equivalents—that nonetheless require immediate invasive evaluation and reperfusion therapy.^5^^,^^6^

Although the Aslanger pattern was initially described mainly in association with left circumflex artery occlusion, subsequent observations indicate that it may also occur in RCA occlusion, particularly when right ventricular ischemia contributes to the injury vector.^4^ In the case presented by López-Tejada et al,^1^ recognition of this subtle but high-risk ECG pattern allowed clinicians to rapidly identify the underlying ischemic mechanism despite the absence of traditional STEMI criteria.

Another important lesson emerging from this report concerns the clinical significance of occlusion involving a nondominant RCA. Because coronary dominance determines the extent of left ventricular myocardium supplied by a vessel, occlusion of a nondominant RCA has historically been considered relatively benign. However, this assumption may underestimate the physiological importance of right ventricular perfusion.

The right ventricle is particularly susceptible to electrical instability during acute ischemia. Even in the absence of extensive left ventricular involvement, right ventricular infarction may precipitate severe ventricular arrhythmias, hemodynamic compromise, or sudden cardiac arrest.^7^ In the present case, occlusion of a relatively small nondominant RCA resulted in profound right ventricular dysfunction and malignant ventricular arrhythmias, culminating in pulseless ventricular fibrillation.^1^

From a practical standpoint, several clinical implications arise from this case. Unexpected hemodynamic deterioration or malignant arrhythmias after aortic root surgery should immediately raise suspicion for coronary compromise. Clinicians should remain familiar with high-risk ECG patterns that may indicate acute coronary occlusion, even in the absence of classic ST-segment elevation. Finally, early recognition of these scenarios should prompt urgent coronary angiography, as timely revascularization may be lifesaving.

The case described by López-Tejada et al^1^ underscores how attentive ECG interpretation can reveal acute coronary occlusion in complex postoperative scenarios. Recognition of the Aslanger pattern reinforces the enduring diagnostic value of the ECG and highlights the importance of identifying high-risk ECG signatures beyond conventional STEMI criteria.

Ultimately, when the ECG speaks—even without classic ST-segment elevation—clinicians must be prepared to listen carefully, as subtle ECG patterns may be the only warning sign of an occluded coronary artery.

## Funding Support and Author Disclosures

The author has reported no relationships relevant to the contents of this paper to disclose.
